# Multi-source UAV remote sensing for cotton Verticillium wilt resistance grading using TPAC-Net

**DOI:** 10.3389/fpls.2026.1911947

**Published:** 2026-09-07

**Authors:** Haoxing Luo, Jiaying Chen, Xinhui Li, Yue Hu, Chaolin Song, Jieyin Chen, He Zhu, Ran Li, Deyong Chen, Yurong Qian

**Affiliations:** 1 Xinjiang Engineering Research Center of Big Data and Intelligent Software, School of Software, Xinjiang University, Urumqi, China; 2 Key Laboratory of Software Engineering, Xinjiang University, Urumqi, China; 3 International Joint Laboratory of Multilingual Cognitive Computing Along the Silk Road, School of Computer Science and Technology, Xinjiang University, Urumqi, China; 4 Xinjiang Key Laboratory of Crop Gene Editing and Germplasm Innovation, Institute of Western Agriculture, Chinese Academy of Agricultural Sciences, Changji, China; 5 State Key Laboratory for Biology of Plant Diseases and Insect Pests, Institute of Plant Protection, Chinese Academy of Agricultural Sciences, Beijing, China; 6 Liaoning Provincial Institute of Economic Crops, Liaoyang, China

**Keywords:** cotton Verticillium wilt, crop breeding, deep learning, multi-source remote sensing, multispectral imagery, plant disease phenotyping, resistance grading, UAV phenotyping

## Abstract

**Introduction:**

Cotton Verticillium wilt resistance grading under field conditions remains challenging because disease symptoms are spatially heterogeneous within plots, local pathological cues are unevenly distributed across high-resolution unmanned aerial vehicle (UAV) orthomosaics, and disease-related responses are not expressed with the same sensitivity or spatial consistency across RGB, raw multispectral (MS), and vegetation-index (VI) representations.

**Methods:**

To address these challenges, this study proposes TPAC-Net, a UAV-based Tri-source Pathological Alignment and Coupling Network for plot-level cotton Verticillium wilt resistance grading, which organizes red–green–blue (RGB) imagery, raw MS imagery, and VI representations derived from MS bands as complementary representation streams of visible canopy structure, band-level spectral responses, and index-enhanced disease-sensitive spectral contrasts. Based on field survey records and the relative disease index (RDI) defined by GB/T 22101.5-2009, two-class and five-class classification settings were constructed, with the standardized five-class task serving as the primary benchmark. TPAC-Net combines source-specific encoders, a Tri-Source Feature Attention Module (TFAM) for adaptive pathological feature coupling, a Cross-Source Semantic Alignment Module (CSAM) for high-level semantic consistency among complementary representations, and a Local Pathogenic Patch Perception strategy for plot-level decision-making from local patches.

**Results:**

The proposed framework achieved F1-scores of 79.20% and 50.37% under the two-class and five-class settings, respectively. Under the primary five-class task, it yielded an absolute F1-score gain of 14.36 percentage points over the best-performing baseline. Localized-response visualization showed that higher model responses were concentrated in selected local canopy regions, although fine-grained discrimination among intermediate resistance grades remained challenging.

**Discussion:**

These results suggest that multi-source UAV remote sensing can support field-survey-derived RDI resistance-grade inference through structured representation alignment, pathological feature coupling, and local evidence aggregation.

## 1 Introduction

Cotton Verticillium wilt, predominantly caused by the soil-borne fungus *Verticillium dahliae*, is one of the most destructive diseases affecting cotton production. The pathogen can persist in soil and colonize the vascular system, resulting in leaf chlorosis, wilting, boll reduction, yield loss, and deterioration of fiber quality (Zhu et al., 2023; Song et al., 2020). In cotton breeding, disease evaluation is required not only to identify infection but also to distinguish the resistance levels of different germplasm materials. Reliable resistance grading is therefore essential for germplasm screening and cultivar selection. However, field resistance evaluation remains difficult because disease expression is spatially heterogeneous within plots, varies gradually across germplasm materials, and may differ only subtly between adjacent resistance categories (Zhang et al., 2025).

Conventional cotton Verticillium wilt resistance evaluation relies on field investigation, in which plant-level disease-severity records are aggregated to calculate the disease index and relative disease index (RDI) of each plot. This procedure provides an agronomically meaningful basis for standardized resistance classification but is labor-intensive and time-consuming when applied to large breeding populations. Efforts have therefore been made to improve the practicality and remote-sensing suitability of disease assessment. For example, Gui et al., (2025) proposed the Eight-Position Grading method to simplify severity investigation and relate the grading result to yield loss. Such studies indicate the need for high-throughput approaches that retain the breeding relevance of field-derived resistance labels.

Low-altitude unmanned aerial vehicle (UAV) remote sensing provides a non-destructive means of acquiring high-resolution observations over large numbers of field plots. Reviews of UAV-based crop phenotyping have demonstrated its value for measuring structural and physiological traits and supporting crop-breeding applications (Xie and Yang, 2020; Tanaka et al., 2024). UAV platforms have also been increasingly used for crop disease detection and monitoring because they can capture spatial and spectral variation in field canopies (Joshi et al., 2024). For cotton Verticillium wilt, temporal airborne hyperspectral observations have been used to evaluate resistance in relation to disease progression (Wang et al., 2025), while UAV multispectral time-series observations have supported high-throughput screening of resistant germplasm (Xu et al., 2025a). These studies establish UAV remote sensing as a useful phenotyping tool. The remaining methodological problem is how to map a high-resolution plot orthomosaic acquired at a single observation date to a standardized, field-survey-derived RDI resistance grade.

Existing remote-sensing studies of cotton Verticillium wilt have mainly addressed disease detection, severity assessment, or disease-index estimation. UAV multispectral features and vegetation indices have been combined with regression and machine-learning models to assess disease severity (Li et al., 2024b). Vegetation indices, color indices, and texture features derived from UAV multispectral-visible imagery have also been integrated to improve disease-index estimation (Ma et al., 2024). In addition, UAV hyperspectral imagery has been used with feature-selection and optimization algorithms for canopy-level severity assessment (Li et al., 2024a), and sensitive spectral features have been identified for Verticillium wilt detection using machine-learning methods (Li et al., 2025). Although these studies have advanced remote-sensing assessment of cotton Verticillium wilt, they largely depend on predefined spectral or texture features and are mainly formulated as detection or regression problems. They therefore provide limited support for end-to-end plot-level classification according to standardized RDI resistance intervals.

Deep learning can learn discriminative representations directly from image data and reduce dependence on manually designed features (Upadhyay et al., 2025). UAV-based deep-learning methods have been investigated for the detection and recognition of crop diseases and pests under field conditions (Zhu et al., 2024). For cotton Verticillium wilt, deep models have also been developed to segment disease symptoms under complex field backgrounds (Xu et al., 2025b). However, many existing disease-recognition models are designed for individual leaves, localized symptoms, or directly annotated images. Their problem setting differs from breeding-oriented resistance evaluation, in which the supervision label is derived from aggregated within-plot field records. Recent reviews have also emphasized the challenges of applying plant-disease recognition models under heterogeneous field conditions (Shafay et al., 2025). A plot-level resistance-grading model must therefore learn from large orthomosaic samples in which disease-relevant evidence is unevenly distributed and individual local patches are not independently annotated.

A further challenge is the joint use of complementary UAV-derived representations. Optical sensing studies indicate that visible and spectral observations describe different aspects of plant disease expression (Pereira et al., 2025). Red–green–blue (RGB) imagery primarily records visible changes in canopy color, texture, and structure, whereas raw multispectral (MS) imagery preserves band-level reflectance responses associated with physiological stress. Vegetation-index (VI) representations transform MS bands into index-enhanced spectral contrasts related to vegetation vigor and disease response. Although VI representations are derived from MS imagery and are not independent sensor observations, recent crop-disease research has shown that RGB, MS, and VI inputs can provide complementary information for disease-index inversion (Deng et al., 2024). Their joint use nevertheless introduces representation, alignment, and fusion challenges (Baltrusaitis et al., 2019; Li et al., 2022). Simple input-level concatenation may not preserve source-specific responses, while uncoordinated high-level features may inadequately represent the correspondence between visible structural damage and latent spectral stress. Effective RDI-based resistance grading therefore requires a model that preserves source-specific information, aligns high-level disease-related semantics, and aggregates discriminative local evidence at the plot level.

To address these challenges, this study proposes TPAC-Net, a Tri-source Pathological Alignment and Coupling Network for plot-level cotton Verticillium wilt resistance grading. TPAC-Net uses source-specific encoders to extract hierarchical representations from RGB imagery, raw MS imagery, and MS-derived VI representations. The Cross-Source Semantic Alignment Module (CSAM) encourages high-level semantic consistency among the complementary representations, whereas the Tri-Source Feature Attention Module (TFAM) adaptively couples same-stage features from channel and spatial perspectives. A Local Pathogenic Patch Perception strategy further aggregates representative local responses into the final plot-level prediction. Through this design, TPAC-Net learns the mapping between multi-source UAV observations and field-survey-derived RDI resistance grades.

The main contributions of this study are as follows:

1. To the best of our knowledge, this study is among the first to formulate cotton Verticillium wilt resistance grading as a UAV-based plot-level deep-learning task grounded in field-survey-derived RDI labels and using RGB imagery, raw multispectral imagery, and MS-derived vegetation-index representations. This formulation links multi-source UAV observations with standardized RDI resistance intervals and provides a breeding-oriented framework for high-throughput resistance screening under field conditions.
2. TPAC-Net was developed as a tri-source pathological alignment and coupling framework for plot-level resistance grading. The model integrates source-specific encoding, high-level cross-source semantic alignment, stage-wise pathological feature coupling, and local disease-evidence aggregation to organize visible structural symptoms, raw spectral physiological responses, and index-enhanced disease-sensitive cues within a unified learning framework.
3. Comprehensive experiments under two-class and five-class settings, source-combination comparisons, coupling-strategy ablations, semantic-alignment analysis, top-*k* aggregation analysis, and localized-response visualization were conducted to evaluate TPAC-Net. The results demonstrate improved RDI-based resistance-grade inference under field conditions, while also revealing that fine-grained discrimination among intermediate resistance grades remains challenging.

## 2 Materials and methods

### 2.1 Data acquisition

#### 2.1.1 Field data collection

**Figure 1:**
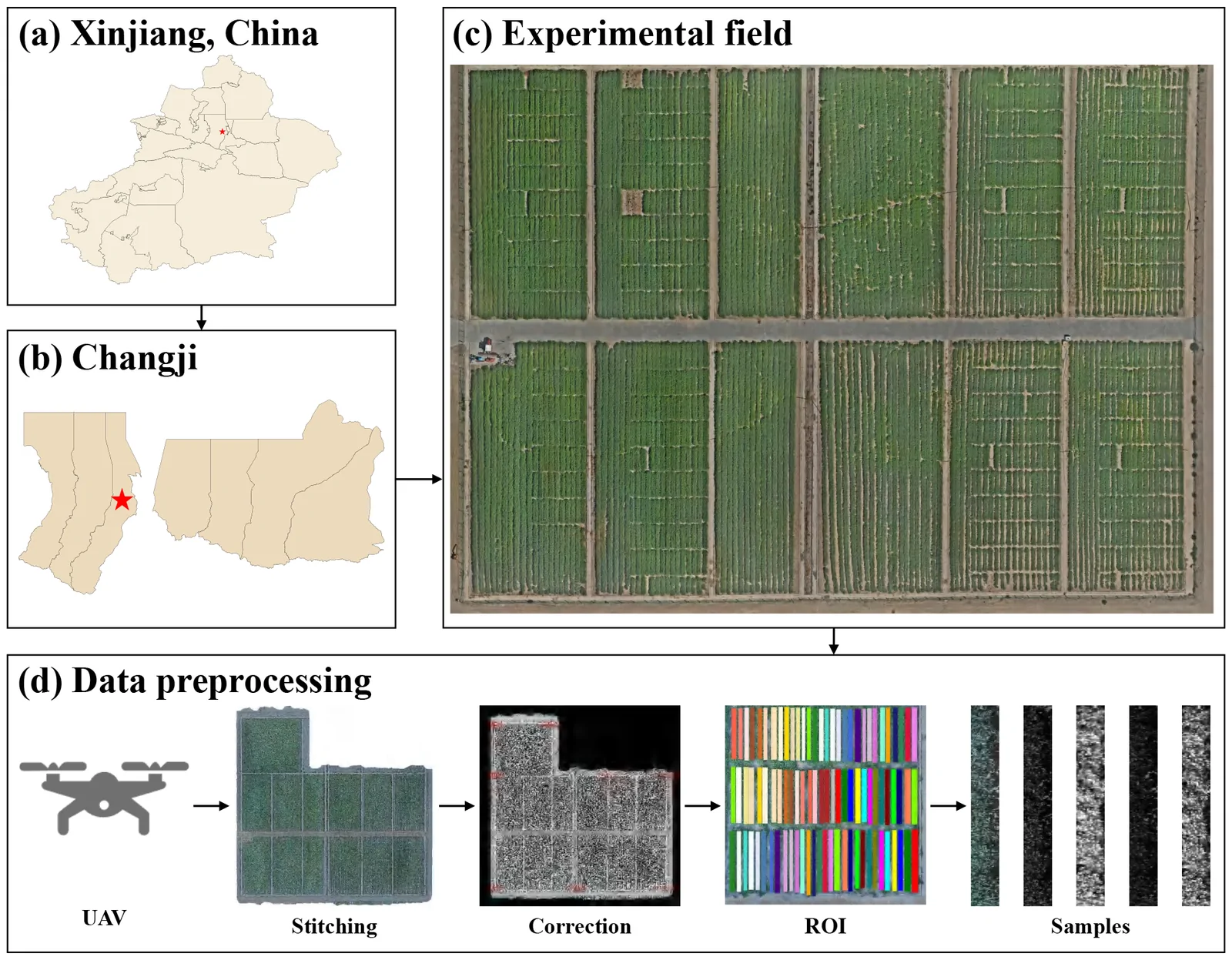
Study site, experimental field, and UAV data-processing workflow. **(a)** Geographic location of the study area in Xinjiang, China. **(b)** Location of the experimental base. **(c)** Overview of the experimental cotton field. **(d)** UAV data-processing workflow from image acquisition to orthomosaic generation, coregistration, vegetation-index construction, and plot-level sample extraction.

The field experiment was conducted at the Changji Integrated Experimental Base, Western Agricultural Research Center, Chinese Academy of Agricultural Sciences, in Dianba Town, Changji City, Xinjiang, China. The experimental field was planted with multiple cotton cultivars for Verticillium wilt resistance evaluation under natural field conditions. The location of the study site and an overview of the experimental field are shown in **Figure 1**.

Field data collection was carried out by plant protection experts on August 22, 2025, during the cotton Verticillium wilt field investigation period. Because this study focused on single-time-point UAV-based resistance grading, the field survey and UAV image acquisition were conducted on the same day to maintain consistency between disease investigation records and remote-sensing observations. The final dataset contained 1,132 valid plot samples used for model development and evaluation.

During field investigation, experts recorded the main agronomic and disease-related information for each plot, including plot identity, serial number, cultivar name, total plant number, diseased plant number, the number of plants at disease severity grades 0–4, disease index (DI), and relative disease index (RDI). Because plant number varied among plots, the total plant number was recorded separately for each plot. A summary of the field survey information is given in **Table 1**.

**Table 1:** Summary of field investigation records used in this study.

Item	Description
Field investigation date	August 22, 2025
Valid plot samples	1,132 plots
Plant number per plot	25–40 plants
Disease severity records	Number of plants at severity grades 0–4 for each plot
Disease assessment records	Diseased plant number, DI, and RDI
Label construction basis	GB/T 22101.5–2009 and RDI-based resistance intervals

#### 2.1.2 UAV RGB and multispectral data acquisition

UAV-based RGB and multispectral data were acquired using a DJI Mavic 3 Multispectral (DJI M3M) platform. The platform integrates a 20 MP RGB camera with a 4/3 CMOS sensor and four 5 MP multispectral cameras. The multispectral cameras provide green, red, red-edge, and near-infrared bands. These data were used to construct spatially corresponding RGB, MS, and VI inputs for plot-level cotton Verticillium wilt resistance grading.

Because the experimental field could not be covered using a single UAV battery, image acquisition was completed in six consecutive flight batches, with the battery replaced between batches. All batches used the same flight altitude, image overlap, and equal-interval capture settings. The raw UAV images from these batches were subsequently processed on the DJI Smart Agriculture platform to generate RGB and multispectral orthomosaic products. A summary of the main UAV acquisition parameters is provided in **Table 2**.

**Table 2:** Main parameters of UAV RGB and multispectral data acquisition.

Parameter	Setting
UAV platform	DJI Mavic 3 Multispectral (DJI M3M)
Acquisition date	August 22, 2025
Flight batches	Six consecutive batches
RGB camera	20 MP, 4/3 CMOS
Multispectral camera	Four 5 MP cameras
MS bands	Green, Red, Red Edge, Near-Infrared
MS band centers	560 ± 16, 650 ± 16, 730 ± 16, and 860 ± 26 nm
Flight altitude	12 m
Flight period	12:00–16:00 (Beijing time)
Acquisition mode	Equal-interval image capture
Side overlap	70%
Forward overlap	80%

### 2.2 Data preprocessing and annotation

#### 2.2.1 Orthomosaic generation, co-registration, and plot clipping

After UAV data acquisition, the raw RGB and multispectral images were processed on the DJI Smart Agriculture platform to generate orthomosaic products, as summarized in the UAV data-processing workflow shown in **Figure 1d**. The generated RGB orthomosaic and multispectral orthomosaic were then imported into ENVI 5.6 with IDL 8.8 (64-bit) for spatial co-registration. RGB imagery was used as the spatial reference, and the multispectral data were aligned to ensure that corresponding pixels from different sources represented the same field location.

After co-registration, plot-level regions of interest (ROIs) were delineated according to the experimental field layout and exported for batch clipping. This process transformed the raw UAV imagery into spatially aligned plot-level RGB and multispectral orthomosaic samples for subsequent tri-source data construction.

**Figure 2:**
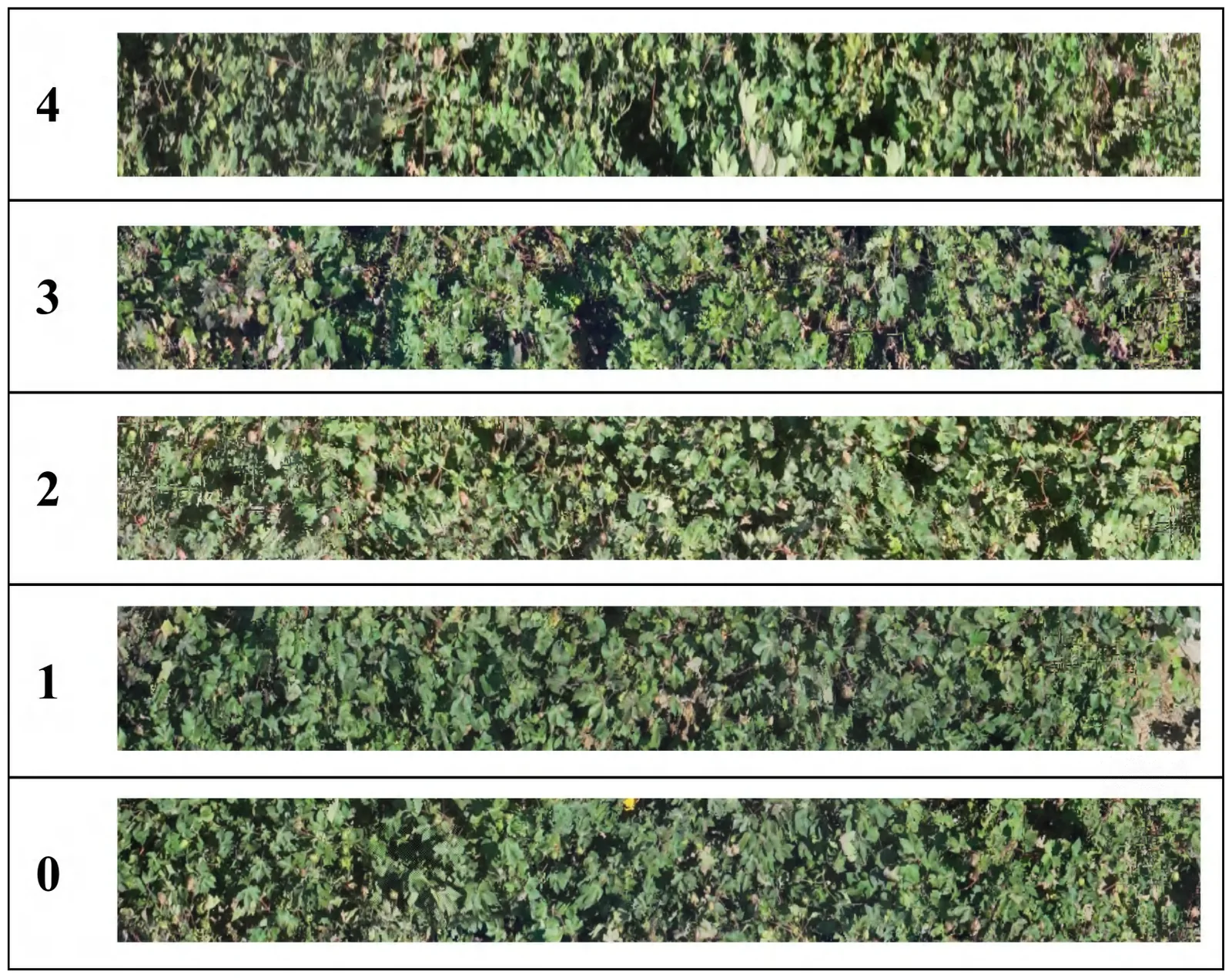
Representative RGB samples from the five RDI-based resistance grades. The labels 0–4 denote immune, highly resistant, resistant, tolerant, and susceptible categories, respectively.

No independent canopy segmentation or background-removal procedure was applied before ROI-based plot clipping, because the ROIs were delineated within the cotton plot regions and the clipped samples were dominated by cotton canopy with limited non-crop background, as illustrated by the representative five-grade samples in **Figure 2**.

#### 2.2.2 Vegetation index computation

Vegetation-index products were calculated from the co-registered multispectral orthomosaic data in ENVI 5.6 with IDL 8.8 (64-bit). Four vegetation indices were used in this study, namely the normalized difference vegetation index (NDVI), which is commonly used to describe canopy vigor and photosynthetically active vegetation (Tucker, 1979); the green normalized difference vegetation index (GNDVI), which enhances sensitivity to canopy greenness and chlorophyll-related variation (Gitelson et al., 1996); the normalized difference red-edge index (NDRE), which emphasizes red-edge responses associated with crop physiological status (Barnes et al., 2000); and the leaf chlorophyll index (LCI), which is related to leaf chlorophyll-sensitive reflectance responses (Datt, 1999).

These indices were selected because cotton Verticillium wilt affects canopy vigor, chlorophyll-related physiological status, and red-edge spectral responses, which can be captured by multispectral and vegetation-index features. Previous cotton Verticillium wilt studies have shown that UAV multispectral and vegetation-index features are useful for disease-severity assessment and monitoring (Li et al., 2024b; Ma et al., 2024). In addition, RGB, MS, and VI representations have been jointly used as complementary inputs for UAV-based disease-index inversion in recent crop disease studies (Deng et al., 2024). Therefore, the selected indices were used as a compact and physiologically interpretable group of disease-sensitive spectral representations, rather than as an exhaustive or empirically optimized vegetation-index set.

The calculated vegetation-index products were clipped using the same plot-level regions of interest as the RGB and multispectral orthomosaics, ensuring spatial correspondence among RGB, MS, and VI samples. Although VI representations were derived from MS bands, they were used as index-enhanced representations rather than independent sensor observations. Raw MS imagery preserves band-level reflectance responses in the green, red, red-edge, and near-infrared bands, whereas VI representations transform these bands into vegetation- and disease-sensitive spectral contrasts. Thus, MS and VI are physically related but encode spectral information in different forms, and they were used together with RGB imagery as complementary input representation streams of TPAC-Net.

#### 2.2.3 Annotation mapping and label construction

Disease labels were assigned according to field survey records and the resistance evaluation criterion defined in GB/T 22101.5–2009 for cotton Verticillium wilt resistance identification. In the field survey, disease severity was first assessed using the standard five-grade scheme (grades 0–4), and the survey results were then used to calculate disease incidence, disease index, and the corrected RDI. In the present study, RDI was used as the unified basis for label construction across all classification settings.

Let *n_i_* denote the number of diseased plants and *n_t_* denote the total number of investigated plants. According to the national standard, the disease incidence rate *R_i_* was calculated using Equation 1:

(1) $$R_{i}=\frac{n_{i}}{n_{t}}\times 100$$

Let *d_c_* denote the disease grade and *n_c_* denote the number of plants at grade *d_c_*. The disease index (DI) was calculated using Equation 2:

(2) $$DI=\frac{\sum (d_{c}\times n_{c})}{n_{t}\times 4}\times 100$$

Because evaluation conditions may vary across regions, years, and batches, the national standard further recommends correcting the disease index using the susceptible check cultivar. Let *DI_CK_* denote the disease index of the susceptible check. The correction coefficient *K* was calculates using Equation 3:

(3) $$K=\frac{50.00}{DI_{CK}}$$

and the relative disease index (RDI) of each tested plot was obtained using Equation 4:

(4) RDI=DI×K

In the present evaluation batch, all selected plots were corrected using the same susceptible check. The disease index of the susceptible check was 40.83, yielding a correction coefficient of *K* = 50.00*/*40.83 ≈ 1.22. The same *K* value was therefore applied to all tested plots in this batch. According to the national standard, evaluation results are considered accurate and reliable when *K* falls within the range of 0.75–1.25.

The primary task of this study was five-class resistance grading, which directly followed the standard RDI intervals. Specifically, plots with *RDI* = 0 were labeled as immune, plots with 0 *< RDI* ≤ 10 as highly resistant, plots with 10 *< RDI* ≤ 20 as resistant, plots with 20 *< RDI* ≤ 35 as tolerant, and plots with *RDI >* 35 as susceptible. For model implementation, these five categories were encoded as class labels 0–4 in ascending order of disease severity.

To provide a coarse disease-screening reference, a supplementary two-class setting was constructed by simplifying the labels into *RDI* = 0 and *RDI >* 0. This setting was used only for comparative evaluation of disease occurrence, whereas the five-class task remained the main resistance grading task throughout the study.

The complete label mapping used in this study is summarized in **Table 3**. For the final dataset used in model development, the numbers of samples in the five standard categories were 597, 252, 115, 78, and 90, respectively. Representative RGB samples from the five RDI-based resistance grades are shown in **Figure 2**. The visual differences among adjacent grades are subtle in the UAV RGB imagery, illustrating the difficulty of fine-grained resistance grading.

**Table 3:** RDI-based label mapping used for the two-class and five-class settings in this study.

Resistance category					
Setting	0 (Immune)	1 (Highly resistant)	2 (Resistant)	3 (Tolerant)	4 (Susceptible)
2-class	*RDI* = 0	*RDI >* 0			
5-class	*RDI* = 0	0 *< RDI* ≤10	10 *< RDI* ≤ 20	20 *< RDI* ≤ 35	*RDI >* 35
No. of samples	597	252	115	78	90

### 2.3 Tri-source pathological alignment and coupling network

**Figure 3:**
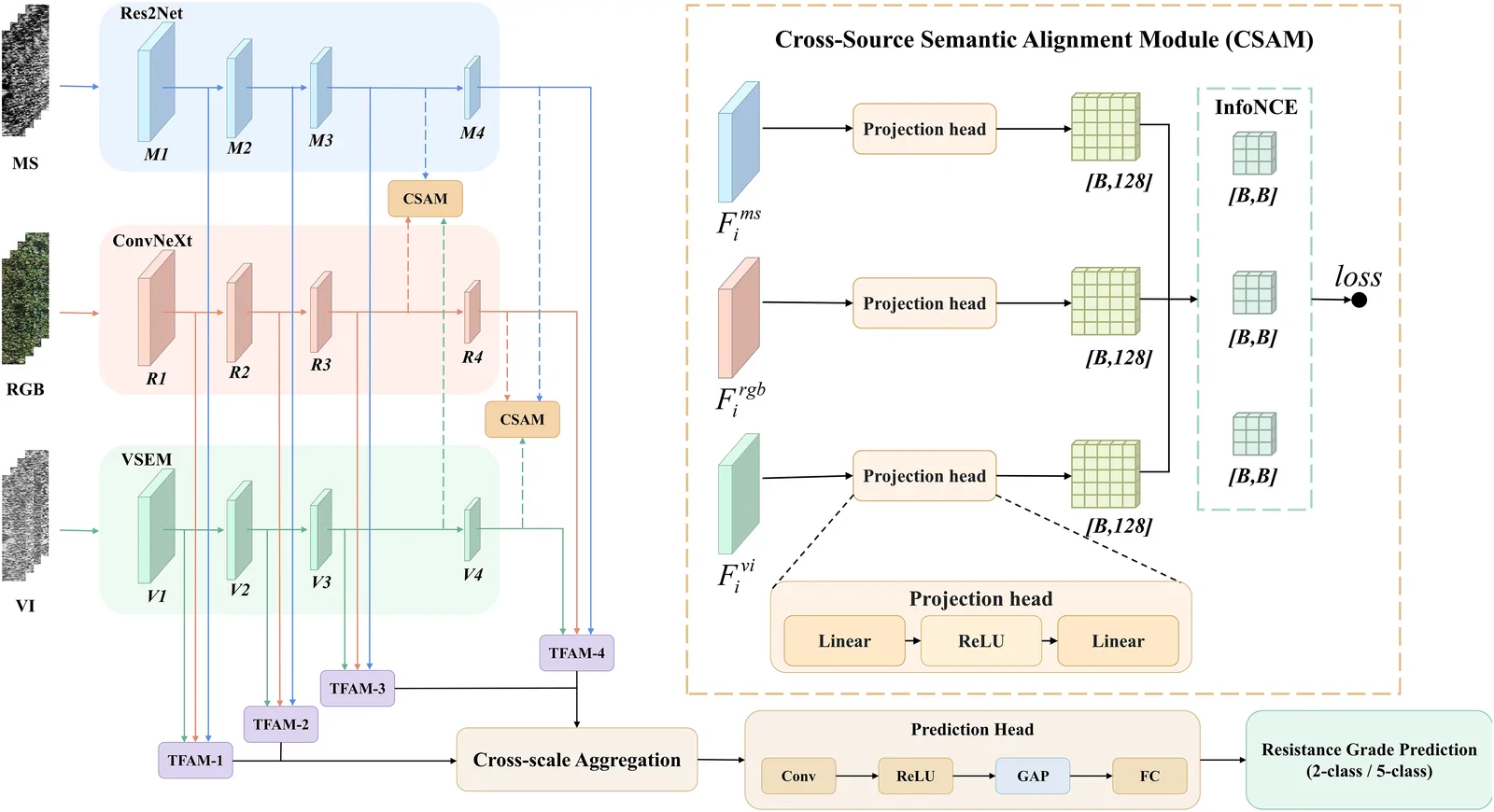
Overall framework of the proposed TPAC-Net for cotton Verticillium wilt resistance grading using RGB, multispectral, and vegetation-index imagery. The framework integrates Local Pathogenic Patch Perception, source-specific encoding, high-level semantic alignment, stage-wise tri-source pathological coupling, cross-scale aggregation, prediction head, and top-*k* mean aggregation for plot-level resistance-grade prediction.

The general framework of the proposed Tri-source Pathological Alignment and Coupling Network (TPAC-Net) is shown in **Figure 3**. It consists of five main components. The first component is the Local Pathogenic Patch Perception strategy, which decomposes each spatially aligned RGB–MS–VI plot sample into local patches and enables image-level weak supervision from plot-level RDI labels. The second component is the source-specific feature extraction module, in which RGB imagery, raw MS imagery, and MS-derived VI representations are encoded by separate branches to extract visible structural features, band-level spectral features, and index-enhanced disease-sensitive features, respectively. The third component is the Cross-Source Semantic Alignment Module (CSAM), which aligns high-level pathological semantics among complementary input representations. The fourth component is the Tri-Source Feature Attention Module (TFAM), which adaptively couples same-stage tri-source features through channel and spatial source-relevance modeling. The fifth component is the cross-scale aggregation and prediction head, which integrates stage-wise coupled features and outputs patch-level logits. The patch-level predictions are then aggregated by top-*k* mean aggregation to obtain the final plot-level resistance-grade prediction. The detailed formulations and implementation of these components are described in the following subsections.

#### 2.3.1 Problem setup and notation

In this study, each clipped orthomosaic sample is regarded as an image, and its RDI-based resistance grade derived from field plot investigation is used as the image-level label. Each image is further decomposed into local patches for model training and prediction. Therefore, the modeling process follows an image-level weak-supervision setting: patch-level inputs are used during learning, while supervision and final evaluation are defined at the image level.

For compact notation, the three model inputs are denoted as shown in Equation 5:

(5) S={ms,rgb,vi}

corresponding to raw multispectral imagery, RGB imagery, and vegetation-index representations, respectively. The image-level label is denoted as *y* ∈ {0,1*,…,C* − 1}, where *C* is the number of classes.

After patch extraction, each image is represented as a set of *M* local patches, as defined in Equation 6:

(6) $$X={\left\{x_{m}\right\}}_{m=1}^{M},\;x_{m}=\{x_{m}^{s}|s\in S\}$$

Here, each local patch contains spatially aligned inputs from the three sources. All local patches inherit the image-level label *y* of their parent image during training.

For source-specific encoding, the feature map extracted from source *s* at stage *i* is denoted as $F_{i}^{s}$, where *s* ∈ S and *i* ∈ {1,2,3,4}. The four stage-wise coupled features generated by TFAM are denoted as ${\left\{F_{i}^{fus}\right\}}_{i=1}^{4}$. In CSAM, P = {(ms,rgb),(ms,vi),(rgb,vi)} denotes the set of source pairs used for high-level semantic alignment. The semantic alignment loss weight is denoted by *λ_mi_*, and the number of selected local patches in image-level aggregation is denoted by *k*.

#### 2.3.2 Local pathogenic patch perception for image-level weak supervision

Cotton Verticillium wilt symptoms are spatially heterogeneous within a plot, and only a subset of local regions may contain representative disease evidence, such as localized chlorosis, partial wilting, vein-related necrosis, or canopy discontinuity. To model this spatial heterogeneity under the image-level supervision defined above, each spatially aligned RGB–MS–VI image is decomposed into local patches. This formulation is related to multiple-instance learning, in which local patches are regarded as instances and the complete plot image provides the supervision label (Ilse et al., 2018).

TPAC-Net therefore treats each patch as a candidate disease-relevant region and learns patch-level responses that are subsequently aggregated into the final plot-level prediction.

For a local patch *x_m_*, TPAC-Net predicts the logits $o_{m}\in {\mathbb{R}}^{C}$ and obtains the class-probability vector by softmax normalization, as shown in Equation 7:

(7) $$p_{m}=\text{Softmax}(o_{m})$$

where $p_{m}=[p_{m}^{\left(0\right)},p_{m}^{\left(1\right)},\ldots ,p_{m}^{\left(C-1\right)}]$ and $p_{m}^{\left(c\right)}$ denotes the predicted probability of class *c*.

Let *w_c_* denote the class weight assigned to class *c*. Using the inherited image-level label *y*, the weighted cross-entropy loss over the local patches of an image is defined as shown in Equation 9

(8) $$L_{cls}=-\frac{1}{M}\sum_{m=1}^{M}\sum_{c=0}^{C-1}w_{c}\;I(y=c)\text{log }p_{m}^{\left(c\right)}$$

where I(·) is the indicator function. Since all local patches inherit the same image-level label, Equation 8 encourages patch representations to learn disease-relevant responses under image-level weak supervision while accounting for class imbalance.

During validation and testing, patch-level predictions from the same image are aggregated into an image-level prediction. For class *c*, the patch-level probabilities are collected as shown in Equation 9:

(9) $$P^{\left(c\right)}=\{p_{1}^{\left(c\right)},p_{2}^{\left(c\right)},\ldots ,p_{M}^{\left(c\right)}\}$$

The top-*k* responses for class *c* are selected according to Equation 10:

(10) $$T^{\left(c\right)}=\text{TopK}(P^{\left(c\right)},k)$$

The image-level probability of class *c* is then computed by top-*k* mean aggregation, as shown in Equation 11:

(11) $${\hat{p}}^{\left(c\right)}=\frac{1}{k}\sum_{p\in T^{\left(c\right)}}p$$

The final predicted label of the image is obtained using Equation 12:

(12) $$\hat{y}=\text{arg }\underset{c\in \{0,1,\ldots ,C-1\}}{\max}{\hat{p}}^{\left(c\right)}$$

In the main experiments, top-*k* mean aggregation is used with *k* = 3. This strategy retains a small number of highly responsive local patches for each class, thereby reducing the influence of weakly informative regions while avoiding excessive dependence on a single maximum response.

#### 2.3.3 Source-specific encoders for complementary pathological representations

Before cross-source semantic alignment and stage-wise feature coupling, TPAC-Net adopts source-specific encoders to preserve the dominant information form of each input representation. This design avoids directly mixing complementary RGB, raw MS, and MS-derived VI representations at the input level, while keeping the four-stage output dimensions consistent for subsequent coupling.

For the RGB branch, the input is three-channel visible imagery, and the main disease-related cues include canopy chlorosis, local wilting, texture degradation, leaf deformation, and structural discontinuity. These cues are primarily spatial and visual. Therefore, a ConvNeXt-based encoder is employed to extract hierarchical RGB features (Liu et al., 2022). ConvNeXt provides a modern convolutional hierarchy with stage-wise feature extraction and strong visual representation capability, making it suitable for learning texture degradation and canopy structural patterns from RGB imagery. The RGB encoder consists of a convolutional stem and four ConvNeXt stages, and its stage-wise outputs are projected to the unified channel dimensions used by the tri-source framework. Given an RGB patch, the branch produces four feature maps ${\left\{F_{i}^{rgb}\right\}}_{i=1}^{4}$, which represent visible pathological manifestations at different semantic levels. For the raw MS branch, the input contains four band-level reflectance channels, including green, red, red-edge, and near-infrared bands. Disease-related physiological stress may appear as reflectance variations at different spatial scales and spectral-response intensities. Therefore, a Res2Net-based encoder is adopted for the MS branch (Gao et al., 2019). Res2Net constructs hierarchical residual-like connections within a single residual block and represents multi-scale features at a more granular level, making it suitable for capturing multi-scale reflectance variations associated with physiological stress. The MS encoder uses a convolution–batch normalization–ReLU stem followed by max pooling and four Res2Net stages. Given an MS patch, the branch produces four stage-wise feature maps ${\left\{F_{i}^{ms}\right\}}_{i=1}^{4}$.

For the VI branch, the inputs are not independent sensor observations but index-enhanced transformations derived from the MS bands. VI maps emphasize vegetation vigor, chlorophyll-related variation, and red-edge disease-sensitive contrasts. Therefore, the VI branch adopts a Res2Net-based encoder equipped with a Vegetation-index Spatial Enhancement Module (VSEM). Res2Net is retained to preserve multiscale index-level representation ability, while VSEM is introduced to emphasize spatially informative disease-sensitive responses in VI maps. Specifically, after the convolution–batch normalization–ReLU stem and max-pooling operation, VSEM is inserted before each of the four Res2Net stages and applies spatial attention to the incoming feature map before hierarchical feature extraction. Each spatial-attention unit generates a single-channel spatial weight map from channel-wise average and maximum responses and reweights the incoming feature map before it enters the corresponding Res2Net stage. This design enables the VI branch to emphasize disease-sensitive index responses at different feature levels while preserving the multi-scale representation ability of Res2Net. Given a VI patch, the branch produces four feature maps ${\left\{F_{i}^{vi}\right\}}_{i=1}^{4}$, which provide index-enhanced disease-sensitive representations derived from MS measurements.

Although different encoder designs are used for the three input sources, the output channel dimensions are unified at each stage to support subsequent stage-wise coupling and avoid arbitrary channel expansion before fusion. In this study, the channel dimensions of the four stages are set as specified in Equation 13:

(13) $$\left[C_{1},C_{2},C_{3},C_{4}]=[32,64,128,256\right]$$

Accordingly, the three encoders jointly generate source-specific stage-wise features summarized in Equation 14:

(14) $$\left\{F_{i}^{s}|s\in S,\;i=1,2,3,4\right\}$$

These hierarchical source-specific features provide the basis for subsequent cross-source semantic alignment and stage-wise pathological feature coupling.

#### 2.3.4 Cross-source semantic alignment module

Because the three input streams encode disease information in different forms, features from the same local patch may still occupy partially inconsistent high-level spaces, especially because visible symptoms and spectral responses differ in sensitivity and spatial expression. For fine-grained RDI-based resistance grading, such inconsistency may weaken the correspondence between visible structural damage and latent physiological stress.

In the tri-branch encoder, shallow features mainly retain source-specific low-level details, such as texture, edge, color, and reflectance variations. Forcing these shallow features into a shared space too early may weaken useful source-specific disease cues. In contrast, stage-3 and stage-4 features contain more abstract pathological semantics related to disease severity and resistance discrimination, and are therefore more suitable for cross-source semantic correspondence learning.

Based on this consideration, TPAC-Net introduces the CSAM at stage 3 and stage 4, as shown in the CSAM branch of **Figure 3**. CSAM projects source-specific high-level features into a shared embedding space and optimizes a symmetric InfoNCE-based cross-source alignment objective (Oord et al., 2018). Through this design, CSAM encourages complementary representations from the same local patch to become semantically closer while keeping representations from different samples separable. The effect of CSAM alignment stages is further examined in the parameter experiments.

For the *i*-th high-level stage, where *i* ∈ {3,4}, the source-specific inputs to CSAM are denoted as shown in Equation 15:

(15) $$F_{i}=\{F_{i}^{s}|s\in S\}$$

Global average pooling (GAP) is first applied to each feature map to obtain compact source-specific semantic descriptors, as defined in Equation 16:

(16) $$G_{i}^{s}=\text{GAP}(F_{i}^{s}),\;s\in S$$

Each descriptor is then mapped into a shared embedding space through a source-specific projection head, as shown in Equation 17:

(17) $$Z_{i}^{s}=P_{s}(G_{i}^{s}),\;s\in S$$

where *P_s_*(·) denotes the projection head of source *s*. Each projection head consists of two fully connected layers with a ReLU activation between them, and the output dimension is set to 128.

After *ℓ*_2_ normalization, pairwise similarity matrices are constructed between different sources within the same mini-batch, as defined in Equation 18:

(18) $$S_{i}^{a,b}=\frac{{\bar{Z}}_{i}^{a}{\left({\bar{Z}}_{i}^{b}\right)}^{⊤}}{\tau },\;(a,b)\in P$$

where $\bar{Z}$ denotes the normalized embedding matrix, τ is the temperature coefficient, and P={(ms,rgb),(ms,vi),(rgb,vi)} denotes the set of source pairs. Each similarity matrix has a size of *B* × *B*, where *B* is the batch size.

For each source pair, the diagonal entries correspond to positive pairs from the same local patch, whereas the off-diagonal entries correspond to negative pairs from different patches in the mini-batch. Let

(19) $$y_{B}={\left[0,1,\ldots ,B-1\right]}^{⊤}$$

denote the identity-matching label vector within a mini-batch, as defined in Equation 19. For a source pair (*a,b*) at stage *i*, the symmetric InfoNCE-based alignment loss is defined in Equation 20:

(20) $$L_{i}^{a,b}=\frac{1}{2}[\text{CE}(S_{i}^{a,b},y_{B})+\text{CE }({\left(S_{i}^{a,b}\right)}^{⊤},y_{B})]$$

where CE(·) denotes the cross-entropy loss. The first term aligns source *a* to source *b*, and the second term aligns source *b* to source *a*.

At each high-level stage, the alignment loss is computed by averaging the losses over all source pairs, as shown in Equation 21:

(21) $$L_{csam}^{\left(i\right)}=\frac{1}{3}(L_{i}^{\text{ms},\text{rgb}}+L_{i}^{\text{ms},\text{vi}}+L_{i}^{\text{rgb},\text{vi}}),\;i\in \{3,4\}$$

The final CSAM loss is obtained by averaging the losses from stage 3 and stage 4, as shown in Equation 22:

(22) $$L_{csam}=\frac{1}{2}\sum_{i\in \left\{3,4\right\}}L_{csam}^{\left(i\right)}.$$

The final training objective combines the patch-level classification loss and the CSAM alignment loss, as shown in Equation 23:

(23) $$L=L_{cls}+\lambda_{mi}L_{csam}$$

where *λ_mi_* controls the contribution of the semantic alignment loss. When *λ_mi_* = 0, the CSAM constraint is removed. In the main five-class experiment, *λ_mi_* is set to 0.2, and the effect of this weight is further examined in the parameter experiments.

CSAM regularizes the stage-3 and stage-4 source-specific representations during training, while the corresponding feature maps are subsequently passed to TFAM for stage-wise coupling.

#### 2.3.5 Tri-source feature attention module

**Figure 4:**
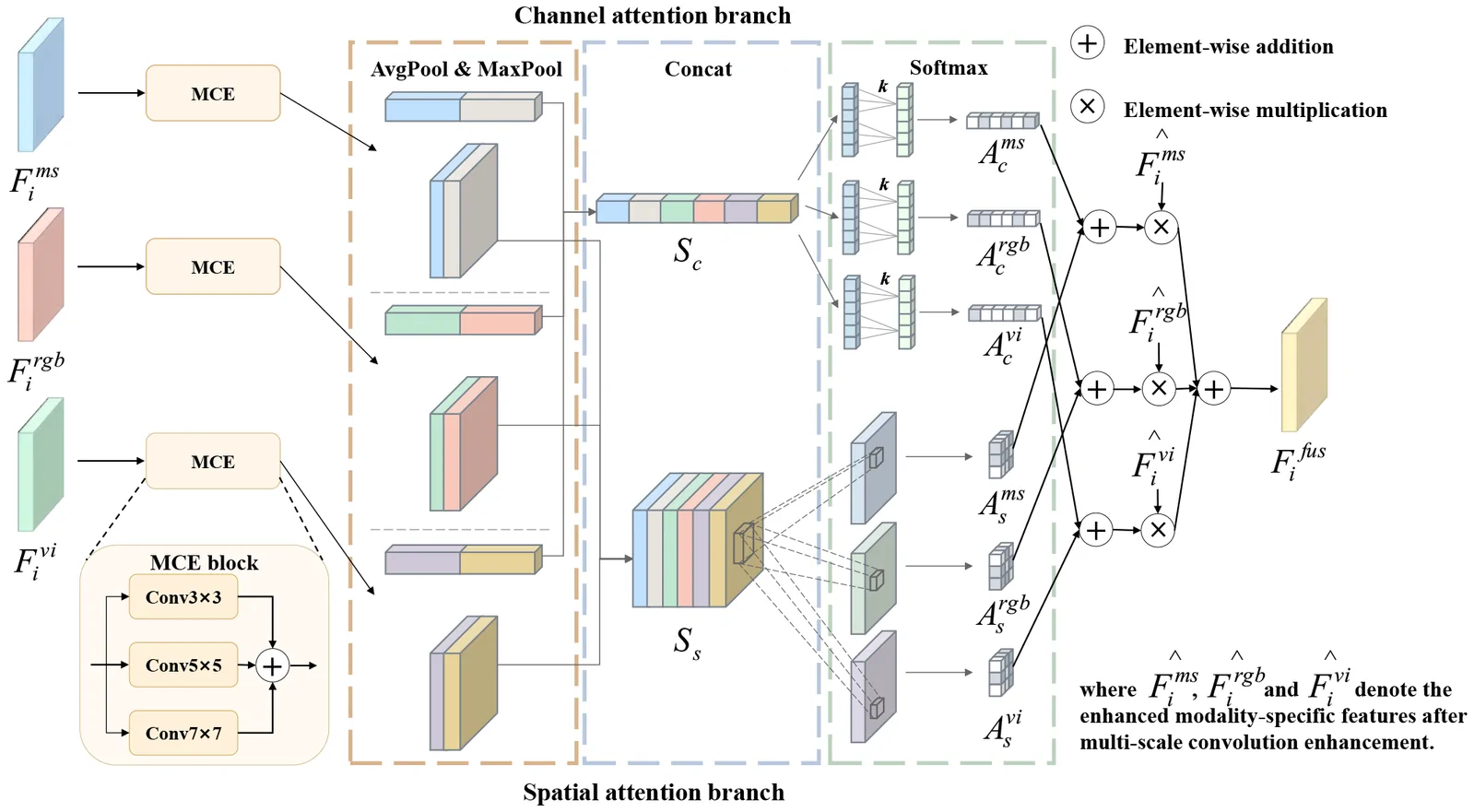
Structure of the proposed Tri-Source Feature Attention Module (TFAM). For each stage, source-specific features are first enhanced by a multi-scale convolution enhancement block and are then adaptively coupled through tri-source channel attention and spatial attention.

As shown in **Figure 4**, TFAM performs stage-wise feature coupling to adaptively integrate RGB, MS, and VI responses at corresponding feature scales. Visible texture degradation, multispectral physiological stress, and index-enhanced disease cues may contribute differently to fine-scale local patches and deeper disease-severity representations. Therefore, TFAM is designed as a stage-wise tri-source coupling module that models source relevance from both channel and spatial perspectives, rather than relying on a fixed fusion rule for heterogeneous features.

At the *i*-th stage, TFAM takes the source-specific feature maps defined above as inputs and performs same-stage tri-source coupling. Each source-specific feature is enhanced by three parallel convolutions with kernel sizes of 3 × 3, 5 × 5, and 7 × 7. The three convolutions use padding to keep their outputs with the same spatial size and channel dimension, so their outputs can be added element-wise. The enhanced feature is computed using Equation 24:

(24) $$\begin{array}{l} {\hat{F}}_{i}^{s}=\;\sigma \left(f_{i}^{3\times 3}\left(F_{i}^{s}\right)\right)+\sigma \left(f_{i}^{5\times 5}\left(F_{i}^{s}\right)\right)\\ \;+\sigma \left(f_{i}^{7\times 7}\left(F_{i}^{s}\right)\right),\;s\in S, \end{array}$$

where $f_{i}^{k\times k}(\cdot )$ denotes the convolution with kernel size *k* × *k* at stage *i*, and *σ*(·) denotes the ReLU activation function.

Based on the enhanced features, TFAM estimates source-wise relevance from two complementary perspectives: channel relevance and spatial saliency. Channel relevance estimates the relative contribution of source-specific pathological responses along the feature-channel dimension, whereas spatial saliency emphasizes disease-relevant local regions across different source observations.

For channel relevance modeling, global average pooling and global max pooling are first applied to each enhanced feature map. The pooled descriptors from the three sources are concatenated to form a channel descriptor defined in Equation 25:

(25) $$D_{ch}=\text{Concat}({\left\{\text{GAP}({\hat{F}}_{i}^{s}),\text{GMP}({\hat{F}}_{i}^{s})\right\}}_{s\in S})$$

where GMP(·) denotes global max pooling. Source-specific one-dimensional convolutional scoring functions are then used to generate unnormalized channel relevance scores defined in Equation 26:

(26) $${\tilde{A}}_{ch}^{s}=g_{ch}^{s}(D_{ch}),\;\;s\in S$$

where $g_{ch}^{s}(\cdot )$ denotes the one-dimensional convolutional scoring function for source *s*. The channel attention weights are obtained by applying softmax normalization across the three sources, as shown in Equation 27:

(27) $$A_{ch}^{s}=\frac{\text{e}\text{x}\text{p}({\tilde{A}}_{ch}^{s})}{\underset{q\in S}{\sum }\text{e}\text{x}\text{p}({\tilde{A}}_{ch}^{q})},\;s\in S$$

where *q* indexes the source in the normalization denominator.

For spatial saliency modeling, average pooling and max pooling are applied along the channel dimension of each enhanced feature map. The spatial descriptors from the three sources are concatenated to form a spatial descriptor defined in Equation 28:

(28) $$D_{sp}=\text{Concat}({\left\{{\text{Avg}}_{c}({\hat{F}}_{i}^{s}),{\text{Max}}_{c}({\hat{F}}_{i}^{s})\right\}}_{s\in S})$$

where Avg*_c_*(·) and Max*_c_*(·) denote average pooling and max pooling along the channel dimension, respectively. Source-specific spatial convolutional scoring functions are then used to generate unnormalized spatial saliency scores defined in Equation 29:

(29) $${\tilde{A}}_{sp}^{s}=g_{sp}^{s}(D_{sp}),\;s\in S$$

where $g_{sp}^{s}(\cdot )$ denotes the spatial convolutional scoring function for source *s*. The spatial attention weights are obtained by applying softmax normalization across the three sources, as shown in Equation 30:

(30) $$A_{sp}^{s}=\frac{\text{e}\text{x}\text{p}({\tilde{A}}_{sp}^{s})}{\underset{q\in S}{\sum }\text{e}\text{x}\text{p}({\tilde{A}}_{sp}^{q})},\;s\in S$$

The final coupled feature of TFAM at stage *i* is defined in Equation 31

(31) $$F_{i}^{fus}=\sum_{s\in S}\left(A_{ch}^{s}+A_{sp}^{s}+\eta \right)⨀{\hat{F}}_{i}^{s},$$

where ⨀ denotes element-wise multiplication, and *η* is a fixed residual constant set to 1 in this study. The residual constant preserves the original source response and reduces excessive suppression caused by attention reweighting.

TFAM is applied separately to the four stages of the tri-branch encoders. Through this stage-wise design, TPAC-Net couples features at the same semantic level rather than mixing all scales indiscriminately. Shallow-stage coupled features mainly support local pathological pattern perception, whereas deeper coupled features contribute to higher-level severity and resistance discrimination. After TFAM produces the four coupled feature maps, TPAC-Net performs cross-scale aggregation before prediction. The coupled features from different stages are first spatially aligned to the resolution of the first-stage feature map by bilinear upsampling and are then concatenated along the channel dimension. The aggregated representation is passed into the prediction head, which consists of a convolution–batch normalization–ReLU block, dropout, global average pooling, flattening, and a fully connected layer. The prediction head outputs patch-level logits, and the corresponding class probabilities are obtained by softmax normalization before image-level top-*k* mean aggregation.

## 3 Experiments

### 3.1 Implementation details

For model development and evaluation, the dataset was split at the image level before patch extraction to avoid information leakage. All patches derived from the same image were kept within the same subset. For each classification setting, the images were divided into training, validation, and test subsets, with both the validation and test ratios set to 0.2. A fixed random seed of 42 was used for data split and model comparison. Channel-wise z-score normalization was applied using statistics estimated only from the training subset, and the same normalization parameters were used for the validation and test subsets.

All images were resized with the short side set to 256 pixels and then cropped into 256×256 local patches using a stride of 64 pixels. During training, RGB, MS, and VI patches were synchronously augmented by random horizontal flipping with a probability of 0.5, random vertical flipping with a probability of 0.5, and random rotation by multiples of 90°. Top-*k* mean aggregation with *k* = 3 was used as the default image-level aggregation strategy. The semantic alignment loss weight was set to *λ_mi_* = 0.2 in the main five-class experiment. The model was optimized using AdamW with a learning rate of 5 × 10^−5^, a weight decay of 5 × 10^−4^, and a gradient clipping threshold of 5.0. The maximum number of training epochs was 80, early stopping patience was 12 epochs, the batch size was 10, and the learning rate was decayed by a factor of 0.5 every 10 epochs after a 5-epoch warm-up. Weighted cross-entropy loss was used for classification, and mixed-precision training with bfloat16 was enabled.

All experiments were implemented in Python 3.10.19 using PyTorch 2.9.1 with CUDA 12.6. The experiments were conducted on a Linux workstation equipped with four NVIDIA L20 GPUs. Under the main five-class setting, the wall-clock training time of TPAC-Net was approximately 2.98 h in this experimental environment. The main Python packages included torchvision 0.24.1, timm 1.0.25, NumPy 2.2.6, pandas 2.3.3, and scikit-learn 1.7.2.

### 3.2 Evaluation metrics

All evaluation metrics were computed after aggregating patch-level predictions into final image-level decisions corresponding to field plots. Accuracy, precision, recall, and F1-score were used to evaluate classification performance. For the five-class setting, precision, recall, and F1-score were reported in macro-averaged form to reduce the influence of class imbalance. F1-score was used as the primary comparison metric because it provides a balanced assessment of precision and recall, while row-normalized confusion matrices were used to analyze class-wise error patterns.

### 3.3 Comparative experiments

The purpose of this experiment was to evaluate the proposed framework from three complementary perspectives: single-source classification performance, tri-source remote-sensing fusion, and the overall benefit of the proposed pathological alignment and coupling framework. The evaluated methods were organized into two groups. The first group contained representative single-source deep backbones trained separately on RGB, MS, or VI inputs, including AlexNet (Krizhevsky et al., 2012), ResNet18 and ResNet50 (He et al., 2016), MobileNetV3-Large (Howard et al., 2019), EfficientNet-B0 and EfficientNet-B3 (Tan and Le, 2019), EfficientNetV2-S (Tan and Le, 2021), ConvNeXt-Tiny (Liu et al., 2022), ConvNeXtV2-Tiny (Woo et al., 2023), and MambaVision-T (Hatamizadeh and Kautz, 2025). These models served as representative single-source baselines. The second group contained LKA-GFNet (Cheng et al., 2026), which was included as a tri-source remote-sensing fusion baseline to evaluate the adaptability of an existing heterogeneous-source fusion strategy to the present resistance-grading task. Together with the input-source ablation and cross-source coupling ablation, these comparisons were designed to separate the effects of backbone capacity, source availability, and fusion strategy.

**Figure 5:**
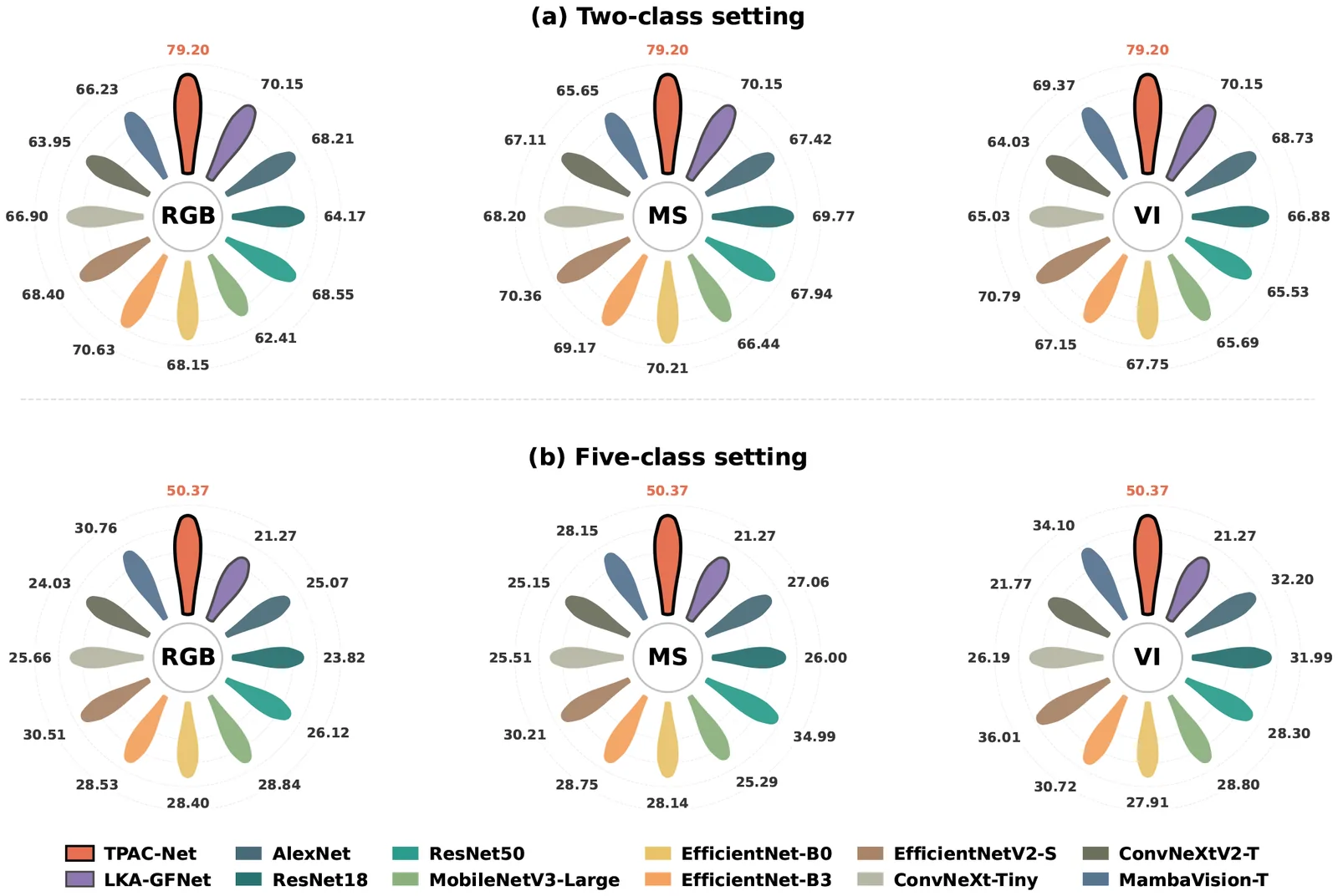
F1-score comparison between TPAC-Net and the baseline models under **(A)** the two-class setting and **(B)** the five-class setting. Single-source backbones were trained separately using RGB, MS, or VI inputs, while LKA-GFNet and TPAC-Net were evaluated using the complete RGB–MS–VI input setting and are repeated in each source panel as tri-source references.

As shown in **Table 4** and **Figure 5**, the proposed framework achieved the best performance under both the two-class and five-class settings. In the two-class task, the best-performing RGB, MS, and VI single-source baselines achieved F1-scores of 70.63%, 70.36%, and 70.79%, respectively, while the tri-source LKA-GFNet baseline achieved 70.15% F1-score. The proposed framework achieved 79.20% F1-score, corresponding to an absolute F1-score gain of 8.41 percentage points over the best-performing baseline. In the five-class task, the best-performing RGB, MS, and VI single-source baselines achieved F1-scores of 30.76%, 34.99%, and 36.01%, respectively, while LKA-GFNet achieved 21.27% F1-score. The proposed framework achieved 50.37% F1-score, corresponding to an absolute F1-score gain of 14.36 percentage points over the best-performing baseline.

**Table 4:** Comparison of baseline models and TPAC-Net under the two-class and five-class settings.

Source	Model	Two-class				Five-class			
		Accuracy (%)	Precision (%)	Recall (%)	F1-score (%)	Accuracy (%)	Precision (%)	Recall (%)	F1-score (%)
RGB	AlexNet	57.52	52.82	96.26	68.21	42.48	23.65	27.16	25.07
RGB	ResNet18	61.95	57.89	71.96	64.17	46.46	23.83	24.92	23.82
RGB	ResNet50	60.62	55.11	90.65	68.55	42.92	25.48	34.16	26.12
RGB	MobileNetV3-Large	55.75	52.20	77.57	62.41	48.23	28.41	30.26	28.84
RGB	EfficientNet-B0	61.95	56.44	85.98	68.15	44.25	27.12	33.40	28.40
RGB	EfficientNet-B3	65.04	58.64	88.79	70.63	42.04	27.61	33.64	28.53
RGB	EfficientNetV2-S	62.39	56.79	85.98	68.40	45.58	29.47	33.72	30.51
RGB	ConvNeXt-Tiny	57.96	53.33	89.72	66.90	43.81	23.76	30.52	25.66
RGB	ConvNeXtV2-Tiny	49.12	48.11	95.33	63.95	34.96	24.28	26.55	24.03
RGB	MambaVision-T	54.87	51.28	93.46	66.23	42.92	31.13	35.13	30.76
MS	AlexNet	61.50	56.25	84.11	67.42	46.02	26.18	31.80	27.06
MS	ResNet18	59.73	54.12	98.13	69.77	46.02	23.38	29.91	26.00
MS	ResNet50	62.83	57.42	83.18	67.94	51.77	35.66	40.45	34.99
MS	MobileNetV3-Large	55.75	51.83	92.52	66.44	41.59	23.21	30.80	25.29
MS	EfficientNet-B0	62.83	56.57	92.52	70.21	42.48	27.89	30.32	28.14
MS	EfficientNet-B3	63.71	57.86	85.98	69.17	45.58	28.53	30.83	28.75
MS	EfficientNetV2-S	66.81	60.96	83.18	70.36	51.33	38.48	35.22	30.21
MS	ConvNeXt-Tiny	57.08	52.53	97.20	68.20	34.51	25.95	32.83	25.51
MS	ConvNeXtV2-Tiny	56.19	52.06	94.39	67.11	37.17	28.00	31.89	25.15
MS	MambaVision-T	60.18	55.48	80.37	65.65	51.77	26.47	32.34	28.15
VI	AlexNet	59.73	54.35	93.46	68.73	42.92	36.66	38.89	32.20
VI	ResNet18	54.42	50.98	97.20	66.88	46.46	30.90	35.16	31.99
VI	ResNet50	64.16	60.16	71.96	65.53	37.17	29.20	32.40	28.30
VI	MobileNetV3-Large	58.41	53.89	84.11	65.69	45.13	28.26	35.14	28.80
VI	EfficientNet-B0	56.19	52.00	97.20	67.75	38.05	32.32	35.03	27.91
VI	EfficientNet-B3	59.73	54.71	86.92	67.15	40.71	30.75	35.13	30.72
VI	EfficientNetV2-S	62.39	55.98	96.26	70.79	50.00	43.09	33.95	36.01
VI	ConvNeXt-Tiny	49.56	48.40	**99.07**	65.03	41.59	25.79	32.73	26.19
VI	ConvNeXtV2-Tiny	51.77	49.49	90.65	64.03	39.38	19.72	29.07	21.77
VI	MambaVision-T	63.27	57.32	87.85	69.37	48.23	34.27	38.33	34.10
Tri-source	LKA-GFNet	64.60	58.39	87.85	70.15	54.42	20.98	25.39	21.27
Tri-source	TPAC-Net	**76.99**	**69.23**	92.52	**79.20**	**61.95**	**53.28**	**50.12**	**50.37**

Values are reported as percentages. Best values are shown in bold, and second-best values are underlined.

These results show that the performance gap cannot be explained by backbone strength or the use of three input representations alone. The inclusion of ConvNeXtV2-Tiny and MambaVision-T broadened the single-source baseline comparison, but neither achieved the best single-source result under the five-class setting. Although LKA-GFNet was designed for tri-source heterogeneous remote-sensing classification, it achieved an F1-score of 21.27% on the present five-class task. One plausible explanation is the difference between its original task setting and the present breeding-oriented resistance-grading problem. LKA-GFNet was developed for tri-source classification of heterogeneous hyperspectral, multispectral, and radar observations, with textual semantic priors and graph-driven decision fusion, whereas the present task involves spatially aligned RGB, raw MS, and MS-derived VI representations under plot-level RDI supervision. In addition, disease evidence is unevenly distributed among local patches, and adjacent RDI grades exhibit subtle differences. TPAC-Net explicitly addresses these characteristics through source-specific encoding, high-level semantic alignment, stage-wise tri-source coupling, and local evidence aggregation. Together with the subsequent ablation results, the comparison suggests that the performance improvement is associated with task-specific representation and coupling mechanisms rather than backbone capacity or the number of input sources alone. Nevertheless, the five-class F1-score of 50.37% also confirms that discrimination among intermediate resistance grades remains challenging.

### 3.4 Ablation experiments

Most ablation experiments were conducted under the five-class setting because it is the primary and more challenging resistance-grading task in this study. Compared with the two-class setting, the five-class task better reflects the standardized RDI-based resistance hierarchy and requires the model to distinguish subtle differences among adjacent resistance levels. Therefore, except for the input-source ablation, which was also reported under the two-class setting for reference, the remaining ablation experiments were performed on the five-class task.

#### 3.4.1 Ablation of input source combinations

The purpose of this experiment was to examine whether resistance grading mainly relied on one dominant source, whether two sources were sufficient, or whether the joint use of RGB, MS, and VI provided additional benefit. Seven input settings were compared: RGB-only, MS-only, VI-only, RGB+MS, RGB+VI, MS+VI, and the full tri-source setting.

As shown in **Table 5**, the full tri-source framework achieved the best performance under both classification settings. In the two-class task, the proposed framework reached 79.20% F1-score, corresponding to absolute F1-score gains of 5.89 and 9.34 percentage points over the best dual-source and single-source settings, respectively. In the five-class task, the proposed framework achieved 50.37% F1-score, corresponding to absolute F1-score gains of 14.92 and 13.36 percentage points over the best dual-source and single-source settings, respectively.

**Table 5:** Ablation of input source combinations under the two-class and five-class settings.

Input setting	Two-class				Five-class			
	Accuracy (%)	Precision (%)	Recall (%)	F1-score (%)	Accuracy (%)	Precision (%)	Recall (%)	F1-score (%)
RGB-only	61.06	55.14	95.33	69.86	56.19	44.74	38.79	36.71
MS-only	53.98	50.73	97.20	66.67	55.31	36.84	42.16	37.01
VI-only	56.64	52.38	92.52	66.89	34.96	28.78	29.69	24.12
RGB+MS	66.81	59.20	96.26	73.31	55.31	34.41	33.93	32.59
RGB+VI	64.16	57.14	97.20	71.97	54.87	36.49	35.90	35.45
MS+VI	60.62	54.69	**98.13**	70.23	50.88	27.10	31.95	26.66
TPAC-Net	**76.99**	**69.23**	92.52	**79.20**	**61.95**	**53.28**	**50.12**	**50.37**

Best values are shown in bold, and second-best values are underlined.

These results indicate that the performance gain of the complete framework cannot be attributed merely to the number of inputs. No individual source or dual-source combination reproduced the performance of the full tri-source setting, and the margin was particularly large in the five-class task. This pattern suggests that the complementary information provided by the three representations becomes increasingly valuable when the model must distinguish subtle differences among adjacent RDI grades.

#### 3.4.2 Effectiveness of TFAM for cross-source coupling

The purpose of this experiment was to determine whether the proposed TFAM was more effective than common cross-source coupling strategies. Under the same tri-source input setting, TFAM was compared with direct concatenation, element-wise summation, weighted summation, channel attention fusion (CAF), and spatial attention fusion (SAF). The comparison was conducted under the five-class task, and only the coupling operation was replaced.

As shown in **Table 6**, TFAM achieved the best overall performance, with 61.95% accuracy and 50.37% F1-score. Direct aggregation methods, including Concat, Sum, and Weighted Sum, produced lower F1-scores of 37.82%, 37.03%, and 35.11%, respectively. CAF and SAF improved the F1-score to 43.83% and 42.22%, respectively, but both remained lower than TFAM.

**Table 6:** Comparison of different cross-source coupling methods on the five-class task.

Model	Accuracy (%)	Precision (%)	Recall (%)	F1-score (%)
Concat	57.96	42.96	37.64	37.82
Sum	56.19	39.55	36.37	37.03
Weighted Sum	50.00	44.84	34.23	35.11
CAF	59.29	41.46	47.63	43.83
SAF	55.75	42.49	46.92	42.22
TFAM	**61.95**	**53.28**	**50.12**	**50.37**

Best values are shown in bold, and second-best values are underlined.

Relative to CAF and SAF, TFAM improved the F1-score by 6.54 and 8.15 percentage points, respectively. It also outperformed the direct aggregation methods, including Concat, Sum, and Weighted Sum. These results indicate that modeling channel-wise source relevance or spatial saliency alone was insufficient to achieve the performance of the complete TFAM. Joint channel–spatial source modeling provided a more effective coupling strategy for the complementary tri-source features in the present five-class task.

#### 3.4.3 Component ablation of CSAM and TFAM

The purpose of this experiment was to clarify the individual and joint contributions of CSAM and TFAM under the five-class task. When TFAM was removed, stage-wise tri-source features were fused by direct concatenation instead of adaptive cross-source coupling. When CSAM was removed, the semantic alignment loss was disabled. The complete TPAC-Net retained both modules.

As shown in **Table 7**, the variant without CSAM or TFAM achieved only 28.63% F1-score, indicating that direct concatenation without semantic alignment or adaptive coupling provided limited grading performance. Enabling CSAM alone improved the F1-score to 37.82%, while enabling TFAM alone achieved 36.64% F1-score. The complete TPAC-Net obtained the best performance, with 61.95% accuracy and 50.37% F1-score.

**Table 7:** Component ablation and complexity analysis of CSAM and TFAM on the five-class task.

Variant	CSAM	TFAM	Acc. (%)	Prec. (%)	Rec. (%)	F1 (%)	Params (M)	FLOPs (G)	Size (MB)
Baseline	No	No	29.56	**53.54**	32.32	28.63	108.57	193.42	414.65
CSAM only	Yes	No	57.96	42.96	37.64	37.82	108.57	193.42	414.65
TFAM only	No	Yes	60.18	40.76	39.11	36.64	99.20	65.89	378.93
TPAC-Net	Yes	Yes	**61.95**	53.28	**50.12**	**50.37**	99.20	65.89	378.93

FLOPs were estimated for a single 256×256 RGB–MS–VI input under the default inference path.

Classification values are reported as percentages. Best values are shown in bold, and second-best values are underlined. Complexity values are reported without ranking.

The matched-complexity comparisons further clarify the contribution of CSAM. Within the concat-based pair, adding CSAM increased the F1-score from 28.63% to 37.82%; within the TFAM-based pair, adding CSAM increased the F1-score from 36.64% to 50.37%. In each pair, the compared variants had identical reported parameter counts, FLOPs, and model sizes. CSAM did not increase the default inference-time FLOPs because its projection heads were used only to construct the semantic alignment objective during training. Therefore, the performance improvement of the complete TPAC-Net was achieved without parameter expansion: compared with the concat-based variants, it used fewer parameters, required substantially fewer FLOPs, and had a smaller model size. Together, these comparisons support the complementary contributions of high-level semantic alignment and adaptive tri-source coupling.

### 3.5 Parameter experiments

#### 3.5.1 Effect of CSAM alignment stage

The purpose of this experiment was to examine the stage-selection strategy of CSAM, namely whether applying CSAM to high-level stages was more effective than aligning shallow, intermediate, or non-adjacent stage features. Seven CSAM settings were compared under the five-class task: Stage 1 only, Stage 2 only, Stage 3 only, Stage 4 only, Stage 1 + 4, Stage 2 + 4, and Stage 3 + 4. The Stage 3 + 4 setting corresponds to the default CSAM configuration used in TPAC-Net.

As shown in **Table 8**, the Stage 3 + 4 setting achieved the best performance, with 61.95% accuracy and 50.37% F1-score. The Stage 4 only setting obtained the second-best F1-score of 43.54%, indicating that deeper semantic representations were more suitable for cross-source alignment than shallow features. In contrast, Stage 1 only and Stage 2 only produced lower F1-scores of 30.88% and 34.71%, respectively, suggesting that shallow features mainly contain low-level texture, color, edge, and reflectance details that are less suitable for strong semantic alignment.

**Table 8:** Effect of CSAM alignment stage on five-class classification performance.

CSAM stage	Accuracy (%)	Precision (%)	Recall (%)	F1-score (%)
Stage 1 only	51.77	31.83	32.49	30.88
Stage 2 only	52.21	38.53	33.48	34.71
Stage 3 only	52.65	38.20	39.04	38.35
Stage 4 only	59.73	49.72	42.28	43.54
Stage 1 + 4	52.21	24.99	31.80	27.67
Stage 2 + 4	57.08	46.32	38.57	39.98
Stage 3 + 4	**61.95**	**53.28**	**50.12**	**50.37**

Best values are shown in bold, and second-best values are underlined.

The non-adjacent stage combinations further support this observation. Stage 1 + 4 achieved only 27.67% F1-score, suggesting that jointly aligning strongly source-specific shallow features and the deepest semantic features may introduce low-level interference into the shared alignment objective. Stage 2 + 4 improved the F1-score to 39.98%, suggesting that intermediate features are more compatible with high-level semantic alignment than Stage 1 features. However, Stage 2 + 4 still remained lower than Stage 4 only and Stage 3 + 4, showing that combining an intermediate stage with the deepest stage did not provide the most effective alignment strategy.

Overall, the comparison supports the design choice of applying CSAM to adjacent high-level stages. Stage 3 and Stage 4 features contain more abstract pathological semantics related to disease severity and resistance discrimination, and their joint alignment provides a more stable semantic basis than shallow-stage or non-adjacent-stage alignment.

#### 3.5.2 Effect of CSAM loss weight

The purpose of this experiment was to examine whether high-level semantic alignment among RGB, MS, and VI representations improves resistance grading, and how strongly this alignment should be imposed. The semantic alignment loss weight *λ_mi_* was varied among 0, 0.1, 0.2, 0.3, and 0.5. When *λ_mi_* = 0, the CSAM constraint was removed.

As shown in **Table 9**, removing the semantic alignment constraint resulted in 36.64% F1-score. Introducing semantic alignment improved performance when the weight was moderate. The best result was obtained at *λ_mi_* = 0.2, where the proposed framework achieved 61.95% accuracy, 53.28% precision, 50.12% recall, and 50.37% F1-score. Compared with *λ_mi_* = 0, the model achieved an absolute F1-score gain of 13.73 percentage points. However, when the alignment weight was increased to 0.5, performance declined to 33.95% F1-score.

**Table 9:** Effect of CSAM loss weight *λ_mi_* on five-class classification performance.

Setting	Accuracy (%)	Precision (%)	Recall (%)	F1-score (%)
$\lambda_{mi}=0$	60.18	40.76	39.11	36.64
$\lambda_{mi}=0.1$	49.56	42.66	36.98	37.76
$\lambda_{mi}=0.2$	**61.95**	**53.28**	**50.12**	**50.37**
$\lambda_{mi}=0.3$	55.31	44.07	40.27	41.34
$\lambda_{mi}=0.5$	55.31	34.55	34.71	33.95

Best values are shown in bold, and second-best values are underlined.

These results indicate that high-level semantic alignment is beneficial when it is appropriately balanced with the primary classification objective. A moderate alignment weight helps associate disease-sensitive cues from the three complementary representations within a shared semantic space, thereby improving resistance discrimination. In contrast, an overly large weight may over-constrain source-specific feature learning and weaken the contribution of the classification objective. Therefore, semantic alignment should guide rather than dominate the overall learning process.

#### 3.5.3 Effect of the top-*k* setting under local pathogenic patch perception

The purpose of this experiment was to examine how many local pathogenic patches should be retained when converting patch-level predictions into an image-level label. Under the top-*k* mean aggregation rule, five values of *k* were tested in the five-class task: 1, 2, 3, 4, and 5.

As shown in **Table 10**, the aggregation performance was sensitive to the value of *k*. When *k* = 1, the model retained only the single highest-response patch for each class, which led to a low F1-score of 31.79%. This setting may overemphasize an isolated local response and is therefore vulnerable to occasional high-confidence but non-representative patches. Increasing *k* to 2 improved the F1-score to 41.95%, indicating that retaining more than one local region is beneficial for plot-level resistance grading. The best performance was obtained at *k* = 3, with 61.95% accuracy and 50.37% F1-score, suggesting that a small set of representative local patches provided the most effective evidence aggregation in this dataset. When *k* was further increased to 4, the F1-score decreased to 46.53%, and increasing *k* to 5 caused a more obvious decline to 32.82%. This decrease indicates that including too many local patches may introduce weakly informative or less disease-relevant regions into image-level prediction.

**Table 10:** Effect of different *k* values in the top-*k* mean image-level aggregation strategy on the five-class task.

k value	Accuracy (%)	Precision (%)	Recall (%)	F1-score (%)
k=1	43.81	30.64	37.90	31.79
k=2	55.75	42.80	41.87	41.95
k=3	**61.95**	**53.28**	**50.12**	**50.37**
k=4	58.85	45.24	49.58	46.53
k=5	49.56	32.66	33.95	32.82

Best values are shown in bold, and second-best values are underlined.

These results show that top-*k* mean aggregation can reduce the influence of weakly informative regions, but the value of *k* needs to balance representative disease evidence and irrelevant local responses. In the present dataset, *k* = 3 provided the best trade-off. However, the sensitivity of performance to *k* also indicates that the aggregation parameter is dataset-dependent and should be regarded as a tunable hyperparameter rather than a fixed universal setting.

## 4 Interpretability analysis

The purpose of the interpretability analysis was to examine whether the learned representations, source-emphasis behavior, class-wise error distributions, and localized response maps were consistent with the pathological rationale of TPAC-Net. Four aspects were analyzed: CSAM semantic alignment, stage-wise source emphasis in TFAM, row-normalized confusion matrices under the two-class and five-class settings, and localized response maps for key disease-relevant patches.

### 4.1 CSAM semantic alignment visualization

To further examine whether CSAM improved high-level cross-source semantic consistency, pairwise cosine similarity matrices of the projected features at Stage 3 and Stage 4 were visualized under two settings: without semantic alignment (*λ_mi_* = 0) and with the best-performing semantic alignment setting (*λ_mi_* = 0.2). The three source pairs, namely MS–RGB, MS–VI, and RGB–VI, were compared. All samples were sorted by class label before visualization to facilitate comparison of class-related cross-source similarity patterns.

**Figure 6:**
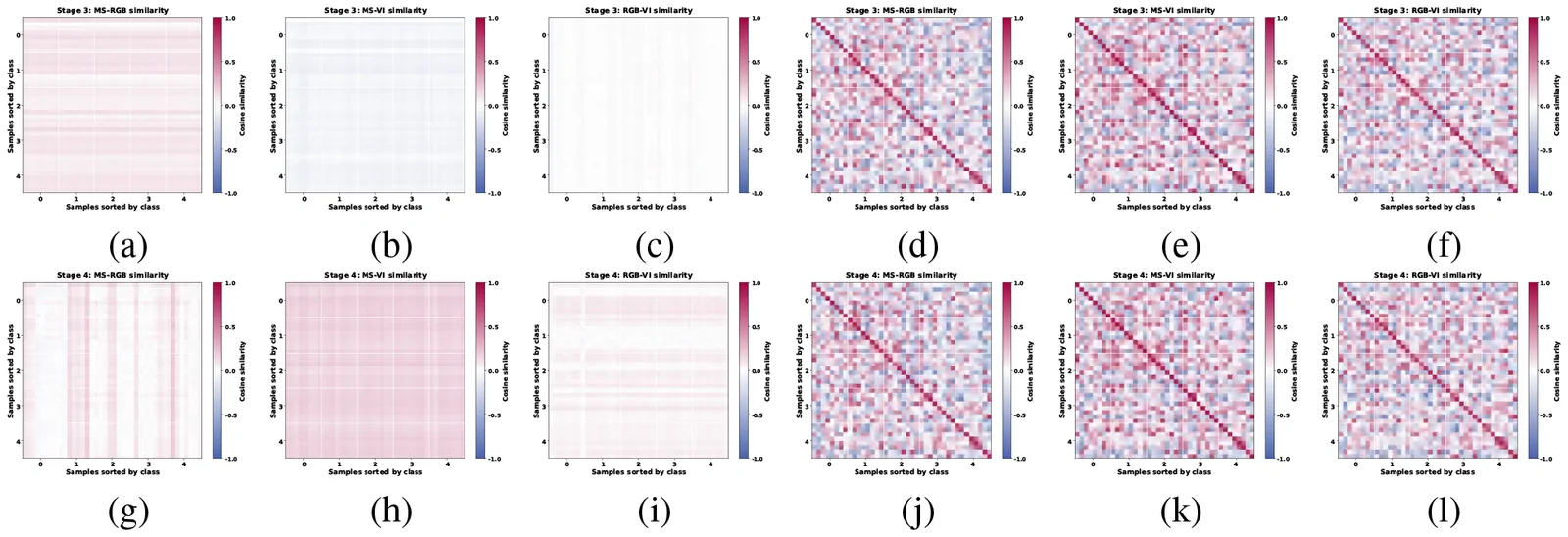
Pairwise cosine similarity matrices of projected features at Stage 3 and Stage 4 under different semantic alignment settings. Panels **(a–c)** show Stage 3 without CSAM, panels **(d–f)** show Stage 3 with CSAM, panels **(g–i)** show Stage 4 without CSAM, and panels **(j–l)** show Stage 4 with CSAM. In each row, the first three panels correspond to *λ_mi_* = 0, and the last three panels correspond to *λ_mi_* = 0.2; within each setting, the source pairs are MS–RGB, MS–VI, and RGB–VI. All samples were sorted by class label before visualization.

As shown in **Figure 6**, the matrices obtained without CSAM show stronger local fluctuations and more irregular positive–negative alternations across both high-level stages and source pairs. After introducing CSAM, the similarity matrices become visually smoother and more homogeneous, with more stable cross-source correspondence after projection. This pattern is consistent with the quantitative improvement observed in the semantic-alignment parameter experiments, supporting the role of CSAM in reducing high-level semantic inconsistency and providing a more coherent semantic basis for subsequent resistance grading.

### 4.2 Effect of stage and source on TFAM attention

**Figure 7:**
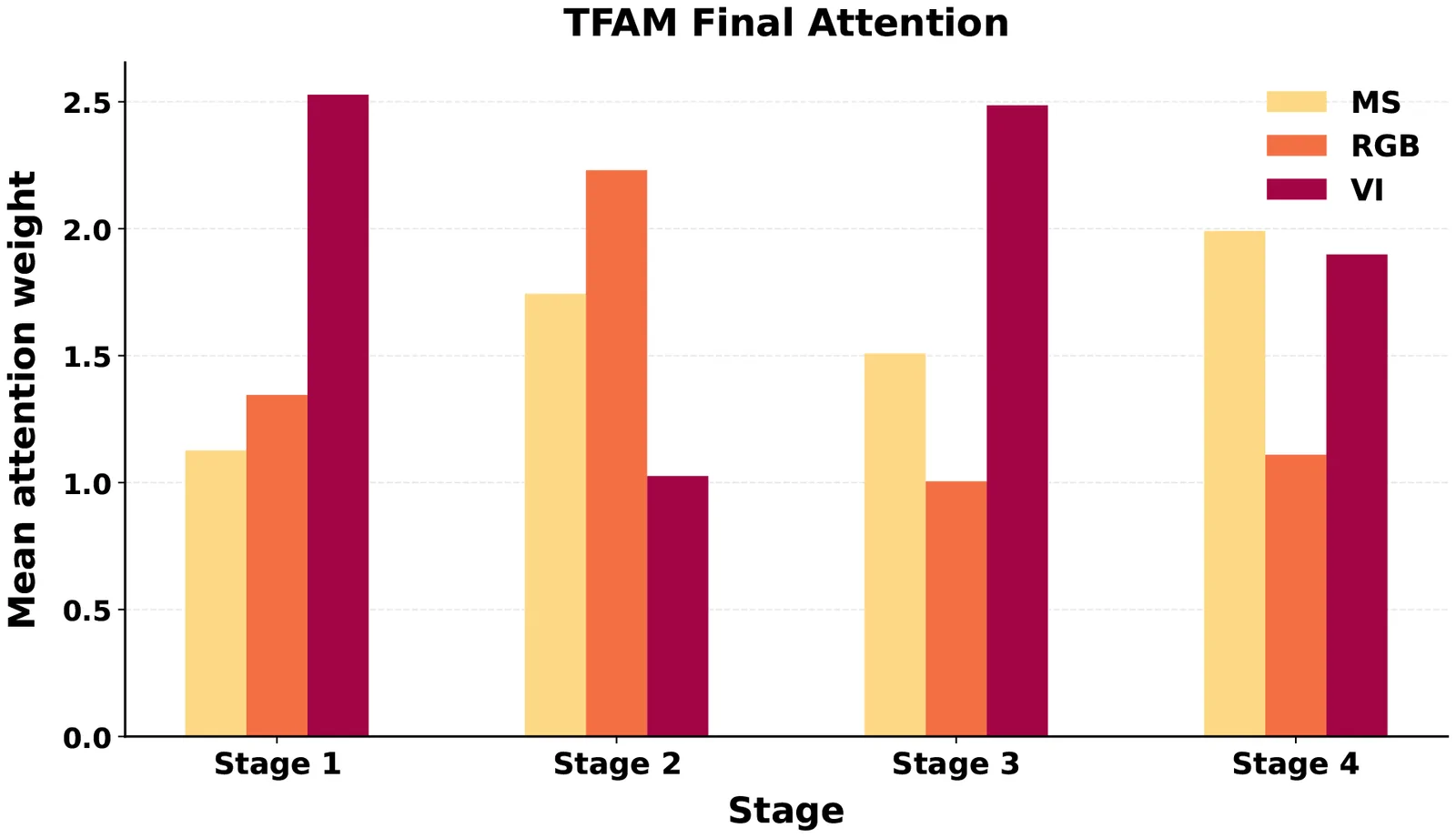
Mean TFAM final attention weights over the test set across the four coupling stages. The relative weighting assigned to MS, RGB, and VI changes with stage and representation level, indicating that TFAM adaptively exploits complementary source information during tri-source pathological coupling.

To further examine how TFAM allocates attention to different sources across stages and representation levels, we visualized the mean final attention weights over the test set at the four coupling stages, as shown in **Figure 7**. A clear stage-dependent preference can be observed. At Stages 1 and 3, the VI source received the highest average attention weight, suggesting that index-enhanced responses contributed strongly at the shallow and intermediate coupling levels. At Stage 2, RGB received the highest average weight, indicating a stronger contribution of visible appearance-related information at this scale. At Stage 4, MS received the highest average weight, while VI remained competitive and RGB received a relatively lower weight, suggesting that deeper coupling placed greater emphasis on multispectral responses associated with disease severity.

Overall, the attention distribution was not uniform across stages. Instead, TFAM dynamically adjusted source emphasis according to feature scale, which supports the design motivation of adaptive tri-source pathological coupling. These results suggest that RGB, MS, and VI features play complementary roles in plot-level resistance grading, and that their relative importance changes with representation depth rather than remaining fixed throughout the network.

### 4.3 Confusion matrix analysis under two-class and five-class settings

**Figure 8:**
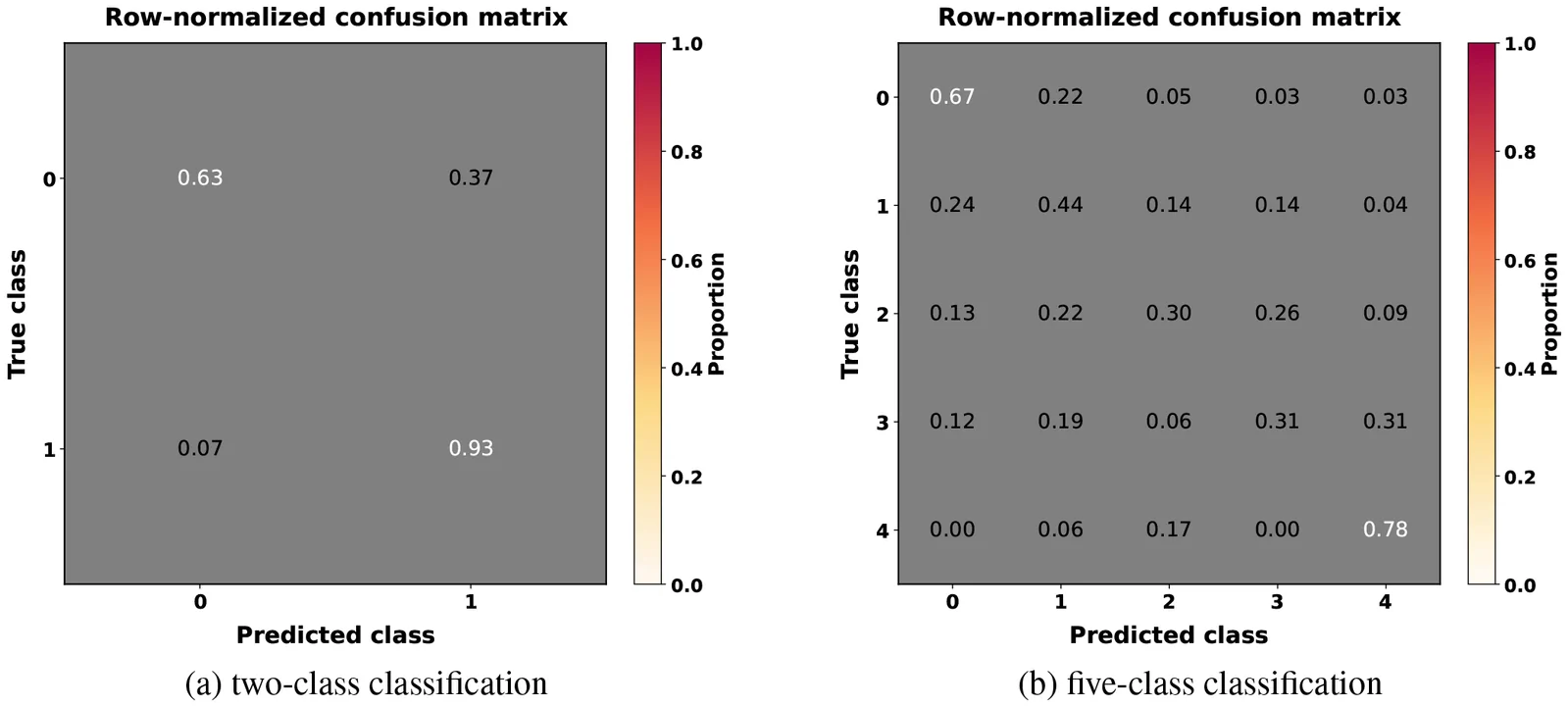
Row-normalized confusion matrices for **(A)** the two-class classification setting and **(B)** the five-class classification setting. Rows denote true labels and columns denote predicted labels. Each cell value represents the proportion of samples in the corresponding true class that were predicted as each class. The two-class task separates immune and diseased plots, while the five-class task follows the RDI-based resistance grading scheme.

To further examine the error patterns under the coarse disease-screening setting and the standardized resistance-grading setting, row-normalized confusion matrices were generated for the two-class and five-class tasks, as shown in **Figure 8**. Each row was normalized by the number of samples in the corresponding true class, and the cell values indicate within-class prediction proportions. Compared with the summary performance reported in the preceding experiments, these matrices provide a more detailed view of how misclassification occurred across disease severity levels.

For the two-class task, the diseased and immune classes achieved class-wise recalls of 0.93 and 0.63, respectively. This indicates that the model was more sensitive to the presence of disease symptoms than to their complete absence. For coarse-grained plot screening, this tendency may be advantageous when failing to identify diseased plots is considered more costly than generating additional false-positive warnings.

The five-class task showed a more complex confusion pattern. The immune and susceptible classes achieved relatively higher class-wise recalls of 0.67 and 0.78, respectively, while the intermediate classes showed stronger confusion. In particular, class 1 was frequently confused with class 0, class 2 was distributed across adjacent classes 1 and 3, and class 3 was partly confused with class 4. These errors were mainly concentrated between neighboring severity levels rather than between distant classes. This pattern is consistent with the ordered RDI-based severity structure, although fine-grained discrimination among transitional resistance levels remains challenging.

### 4.4 Localized response analysis of key disease-relevant patches

**Figure 9:**
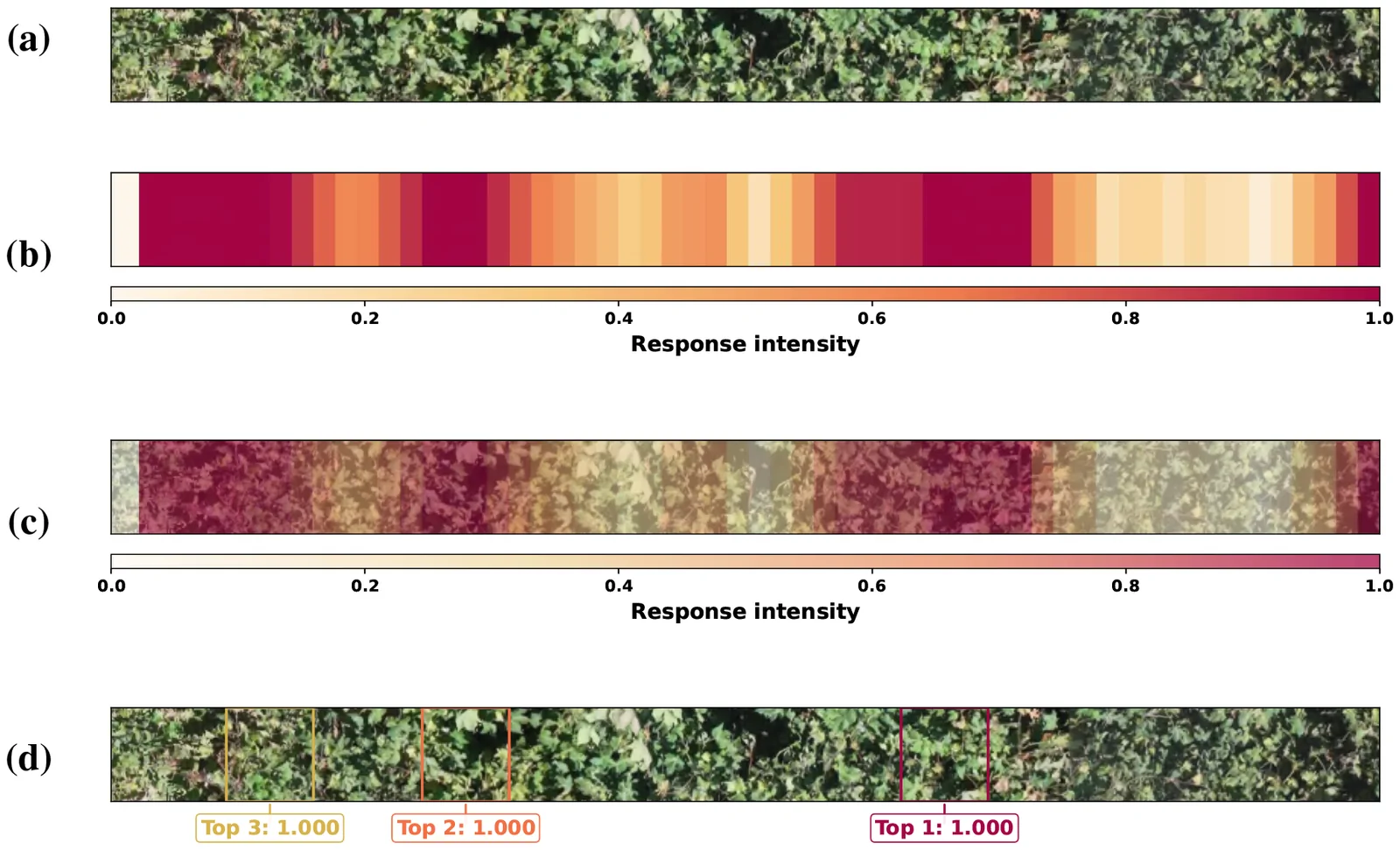
Localized response visualization for a representative plot in the five-class task. **(a)** RGB orthomosaic. **(b)** Localized response map. **(c)** RGB–response overlay. **(d)** Selected top-*k* key patches used for image-level aggregation.

To further examine the spatial response behavior of TPAC-Net, a localized response analysis was conducted on a representative plot from the five-class task. As shown in **Figure 9**, the RGB orthomosaic, localized response map, RGB–response overlay, and selected top-*k* key patches are presented together to provide qualitative visual evidence of how local regions contributed to the image-level prediction.

The localized response map shows spatially heterogeneous model responses across the representative plot. Relatively stronger responses are concentrated in several local regions, while other areas exhibit comparatively weaker activation. When these responses are projected back onto the RGB orthomosaic, the highlighted regions correspond mainly to locally heterogeneous canopy areas with apparent color and texture variations, suggesting that the model tends to emphasize disease-relevant local evidence rather than regions with weaker disease-related manifestations.

In addition, the top-*k* key patches selected during image-level aggregation are all located in the high-response regions of the plot. This observation provides intuitive support for the effectiveness of the Local Pathogenic Patch Perception strategy. In particular, it indicates that the top-*k* mean aggregation mechanism can emphasize discriminative local responses instead of treating all patches equally.

Taken together, this case study complements the semantic-alignment, source-attention, and confusion-matrix analyses by providing spatial evidence of the model response behavior. The high-response regions and selected top-*k* patches were generally located in visually heterogeneous canopy areas, supporting the design rationale that plot-level resistance grading should emphasize representative local regions rather than treating all parts of an elongated orthomosaic as equally informative. Because the available field survey provided plot-level RDI labels, the present analysis focuses on the spatial consistency between model responses, selected patches, and visible canopy heterogeneity at the plot scale. Future studies could further strengthen this analysis by establishing field-to-image annotation protocols that link UAV orthomosaic regions with plant-level symptom observations or quantitative pathological measurements.

## 5 Discussion

The experimental results demonstrate the feasibility of learning field-survey-derived RDI resistance grades from multi-source UAV orthomosaics. TPAC-Net achieved 79.20% F1-score in the supplementary two-class task and 50.37% in the primary five-class task. The substantial performance difference between the two settings indicates that detecting disease occurrence and distinguishing standardized resistance grades are fundamentally different levels of difficulty. The confusion matrices further show that errors in the five-class task were concentrated mainly among adjacent intermediate categories, whereas the immune and susceptible categories were identified more reliably. This pattern is consistent with the gradual variation of RDI and the subtle phenotypic transitions between neighboring resistance intervals. Compared with previous cotton Verticillium wilt studies that mainly focused on disease-severity or disease-index estimation (Li et al., 2024b; Ma et al., 2024; Li et al., 2024a), the present study directly addresses plot-level classification according to standardized RDI resistance grades, thereby extending UAV-based disease assessment toward breeding-oriented resistance evaluation.

The comparative and ablation experiments clarify why the complete TPAC-Net configuration was effective. The full tri-source setting outperformed all evaluated single- and dual-source combinations, indicating that RGB, raw MS, and MS-derived VI representations provided complementary evidence for resistance discrimination. TFAM achieved higher performance than direct aggregation, CAF, and SAF, showing the benefit of jointly modeling channel-wise source relevance and spatial saliency. CSAM further improved the F1-score under matched model complexity, while the complete TPAC-Net required fewer parameters and substantially lower FLOPs than the concat-based variants. These findings demonstrate that the performance gain was associated with the proposed source-specific encoding, high-level semantic alignment, and adaptive stage-wise coupling mechanisms rather than with simple parameter expansion. In addition, the top-*k* experiment showed that plot-level prediction benefited from aggregating a small set of representative local responses, which is consistent with the spatial heterogeneity of Verticillium wilt symptoms within elongated orthomosaic samples.

The present study also identifies several directions for further development. The five-class results show that intermediate resistance grades remain the main source of classification error, partly because their visual and physiological differences are subtle and their sample numbers are relatively limited. Increasing the representation of these categories would provide a stronger basis for fine-grained resistance learning. In addition, the current dataset was collected at one experimental site and one key observation date. Future work will extend the framework to multi-year, multi-site, and multi-temporal observations, examine prediction uncertainty across repeated experiments, and establish finer field-to-image annotations for linking localized UAV responses with plant-level disease observations.

## 6 Conclusion

This study proposed TPAC-Net, a tri-source pathological alignment and coupling framework for plot-level cotton Verticillium wilt resistance grading using UAV-derived RGB imagery, raw multispectral imagery, and MS-derived vegetation-index representations. By integrating source-specific encoding, high-level cross-source semantic alignment, stage-wise tri-source feature coupling, and Local Pathogenic Patch Perception, TPAC-Net was designed to learn field-survey-derived RDI resistance grades from spatially heterogeneous plot-level UAV orthomosaics.

Under the primary five-class setting, TPAC-Net achieved 61.95% accuracy and 50.37% F1-score, corresponding to an absolute F1-score gain of 14.36 percentage points over the best-performing baseline. Together, the comparative, source-combination, coupling-strategy, component-ablation, and parameter experiments indicate that the performance gain was associated with structured tri-source representation learning rather than simple source accumulation or parameter expansion. The interpretability analyses further illustrated high-level cross-source correspondence, stage-dependent source emphasis, class-wise error patterns, and localized model responses.

Overall, this study provides a task-specific framework for linking multi-source UAV observations with standardized field-survey-derived RDI labels, supporting the development of high-throughput resistance-screening tools for cotton breeding.

## Data Availability

The datasets generated and analyzed during the current study are not publicly available due to ongoing related research and institutional data-use restrictions. The data include UAV RGB and multispectral imagery, vegetation-index products, plot-level samples, and field-survey-derived disease/resistance labels. Requests to access the datasets should be directed to the corresponding authors and will be considered upon reasonable request and with permission from the relevant research institutions. Requests to access the datasets should be directed to Deyong Chen, chendeyong@caas.cn; Yurong Qian, qyr@xju.edu.cn.
